# *Porphyromonas gingivalis* induces entero-hepatic metabolic derangements with alteration of gut microbiota in a type 2 diabetes mouse model

**DOI:** 10.1038/s41598-021-97868-2

**Published:** 2021-09-15

**Authors:** Yoichiro Kashiwagi, Shunsuke Aburaya, Naoyuki Sugiyama, Yuki Narukawa, Yuta Sakamoto, Masatomo Takahashi, Hayato Uemura, Rentaro Yamashita, Shotaro Tominaga, Satoko Hayashi, Takenori Nozaki, Satoru Yamada, Yoshihiro Izumi, Atsunori Kashiwagi, Takeshi Bamba, Yasushi Ishihama, Shinya Murakami

**Affiliations:** 1 Department of Periodontology, Division of Oral Biology and Disease Control, Osaka University Graduate School of Dentistry, 1-8 Yamadaoka, Suita, Osaka 565-0871 Japan; 2 Division of Metabolomics, Medical Institute of Bioregulation, Kyushu University, Higashi-ku, Fukuoka, 812-8582 Japan; 3 Graduate School of Pharmaceutical Sciences, Kyoto University, Sakyo-ku, Kyoto, 606-8501 Japan; 4 Division for Interdisciplinary Dentistry, Osaka University Dental Hospital, 1-8 Yamadaoka, Suita, Osaka Japan; 5 Kusatsu General Hospital, Kusatsu, Shiga 525-8585 Japan

**Keywords:** Periodontitis, Type 2 diabetes, Microbiome, Biofilms, Proteomics, Metabolomics

## Abstract

Periodontal infection induces systemic inflammation; therefore, aggravating diabetes. Orally administered periodontal pathogens may directly alter the gut microbiota. We orally treated obese *db*/*db* diabetes mice using *Porphyromonas gingivalis* (*Pg*). We screened for *Pg*-specific peptides in the intestinal fecal specimens and examined whether *Pg* localization influenced the intestinal microbiota profile, in turn altering the levels of the gut metabolites. We evaluated whether the deterioration in fasting hyperglycemia was related to the changes in the intrahepatic glucose metabolism, using proteome and metabolome analyses. Oral *Pg* treatment aggravated both fasting and postprandial hyperglycemia (*P* < 0.05), with a significant (*P* < 0.01) increase in dental alveolar bone resorption. *Pg*-specific peptides were identified in fecal specimens following oral *Pg* treatment. The intestinal *Pg* profoundly altered the gut microbiome profiles at the phylum, family, and genus levels; *Prevotella* exhibited the largest increase in abundance. In addition, *Pg*-treatment significantly altered intestinal metabolite levels. Fasting hyperglycemia was associated with the increase in the levels of gluconeogenesis-related enzymes and metabolites without changes in the expression of proinflammatory cytokines and insulin resistance. Oral *Pg* administration induced gut microbiota changes, leading to entero-hepatic metabolic derangements, thus aggravating hyperglycemia in an obese type 2 diabetes mouse model.

## Introduction

Human oral biofilm-forming bacteria cause chronic inflammatory periodontal infection due to a bacterial symbiosis disorder caused by inadequate oral hygiene^1,2^. *Porphyromonas gingivalis* (*Pg*) causes significant changes in both the amount and composition of normal oral microbiota*. Pg* is a key species causing periodontitis in combination with the other bacteria in periodontal pockets^3,4^. The main cause of periodontal disease is a dental biofilm composed of periodontal bacteria; the accumulation of this biofilm leads to inflammation and destruction of the periodontal tissues. The previous reports demonstrated that diabetes increases the risk of developing destructive periodontal disease about threefold^5^, and periodontal treatment could be important for effective glycemic management in people with type 2 diabetes^6^. Thus, periodontal disease and systemic diseases especially diabetes have a bidirectional influence on each other. Inflammatory cytokines are chronically overexpressed in accelerated periodontitis, which could exacerbate systemic metabolic diseases^7–9^.

The clinical association between periodontal diseases and the poor glycemic control in diabetes is actively investigated^7^. Diabetes is a major risk factor for periodontal disease, with a threefold-higher prevalence of periodontitis in diabetic patients, compared to that in non-diabetic subjects; and poor glycemic control could trigger and worsen periodontitis in diabetes^10–12^. Advanced stages of periodontitis may further impair glycemic control in diabetic patients^13^. The mechanisms connecting these conditions has not been elucidated; however, it is speculated that local infection by oral pathogens, and the release of inflammatory cytokines into blood vessels, could explain the systemic effects of periodontal disease^7–9,14^.

However, recent studies have proposed that the dissemination of periodontal pathogens into the intestinal tract may induce systemic inflammation, metabolic changes, and fatty liver disease in non-diabetic mice models^15,16^. Clarifying this requires identification of orally administered periodontal bacteria in fecal specimens. Bacterial cells and their genomic DNA have not been previously identified in fecal specimens, probably because of their rapid digestion by intestinal enzymes^15,16^.

Here, we studied the presence of *Pg* in fecal specimens at the peptide level, using proteomic analysis of *Pg*-specific peptide fragments, following the oral administration of *Pg* in obese type 2 diabetes mice. Oral bacteria mixed with saliva and food can survive in the acidic stomach environment, and subsequently be transmitted to the intestinal tract with food^17^.

Oral administration of *Pg* in diabetic mice aggravated both fasting and postprandial hyperglycemia, and increased alveolar bone reabsorption. Excessive hepatic gluconeogenesis contributes to hyperglycemia in poorly controlled diabetes^18,19^. Therefore, using genomic, proteomic, or metabolomic analyses, we investigated whether *Pg*-administration increased the mRNA and protein expression of hepatic gluconeogenesis-related enzymes, levels of intrahepatic glucose, and that of lipid metabolites in diabetic mice. Previous studies used metagenome analysis to study the changes in the gut microbiota of *Pg-*treated diabetic mice^15,20^; in this study, we applied metaproteome analysis for the elucidation.

## Results

### Increased fasting and postprandial hyperglycemia following *Pg* treatment

Blood glucose levels under ad libitum feeding in *Pg*-administered mice was significantly (*P* < 0.05) higher than that in control mice after 4 weeks of oral *Pg* treatment without any differences in both food intake, body wight and blood glucose at ad libitum feeding during 4-week period between 2 groups (Table 1). Blood glucose levels at both fasting and 2 h after glucose loading were also significantly higher (*P* < 0.05) in *Pg*-treated mice, compared to control *db*/*db* mice; however, their fasting hyperinsulinemia and serum triglyceride levels were not different between the 2 treatment groups (Tables 2, 3). The intraperitoneal insulin tolerance test showed a similar impaired glucose reduction in the early phase (30 and 60 min after insulin loading) between the 2 treatment groups, indicating that the insulin resistance did not deteriorate further with the *Pg* treatment (Table 3).

**Table 1:** Food intake, body weight and blood glucose at ad libitum feeding in *Porphyromonas gingivalis* (*Pg*)- and carboxymethyl cellulose control-treated *db*/*db* mice fed ad libitum, at baseline and 1–4 weeks after treatment.

	Group	Pre	1 W	2 W	3 W	4 W
Food intake/cage (g)	Control	205 ± 3	208 ± 11	217 ± 4	220 ± 26	204 ± 9
*n* = 4	*Pg*	205 ± 4	211 ± 10	199 ± 38	208 ± 33	202 ± 38
Body weight (g)	Control	35.3 ± 2.8	38.0 ± 2.0	40.3 ± 2.7	42.4 ± 1.6	43.9 ± 1.8
*n* = 8	*Pg*	35.0 ± 2.7	37.7 ± 2.5	40.1 ± 2.5	41.5 ± 2.2	43.0 ± 2.7
Blood glucose (mg/dl)	Control	524 ± 56	615 ± 71	626 ± 119	630 ± 44	651 ± 51
*n* = 8	*Pg*	538 ± 54	619 ± 95	698 ± 44	706 ± 66*	735 ± 73*

Data are expressed as mean ± standard error of mean.

Blood glucose levels were measured at ad libitum feeding.

*P < 0.05 as compared with that of the control.

**Table 2:** Blood glucose and serum insulin levels at fasting condition, and serum triglyceride levels at ad libitum* feeding* condition in *Porphyromonas gingivalis* (*Pg*)- and CMC-treated *db*/*db* mice.

	Blood glucose (mg/dL: fasting)	IRI (μU/mL: fasting)	Triglyceride (mg/dL: ad libitum feeding)
Control	346 ± 124	32.5 ± 18.7	290 ± 101
*Pg*	467 ± 124*	36.5 ± 18.7	252 ± 90

Glucose and insulin levels were measured 3 weeks after treatment, and triglyceride levels 4 weeks after treatment. Data are expressed as mean ± standard error of mean.

IRI: immunoreactive insulin.

*P < 0.05 compared with the control; *n* = 8.

**Table 3:** Oral glucose tolerance test (OGTT) after 10 h overnight fast, and intraperitoneal insulin tolerance test (ipITT) after 1 h fast.

	Gloup	Blood glucose levels (mg/dl)				Area under curve
		0 min	30 min	60 min	120 min	
OGTT	Control	346 ± 124	618 ± 143	577 ± 158	317 ± 138	975 ± 118
	*Pg*	467 ± 124*	642 ± 148	549 ± 138	442 ± 128*	1070 ± 114
IpITT	Control	100 ± 0	76.6 ± 24.2	63.6 ± 19.0	75.3 ± 23.9	158 ± 45
	*Pg*	100 ± 0	75.6 ± 16.4	68.1 ± 13.5	57.9 ± 16.9*	150 ± 30

One-way ANOVA with Tukey’s post hoc test was performed for food intake, body weight, blood glucose level, OGTT, and ipITT. Data are expressed as the mean and SEM. *n* = 8, *P < 0.05 versus CMC control.

### Increased alveolar bone resorption following *Pg* treatment

**Figure 1:**
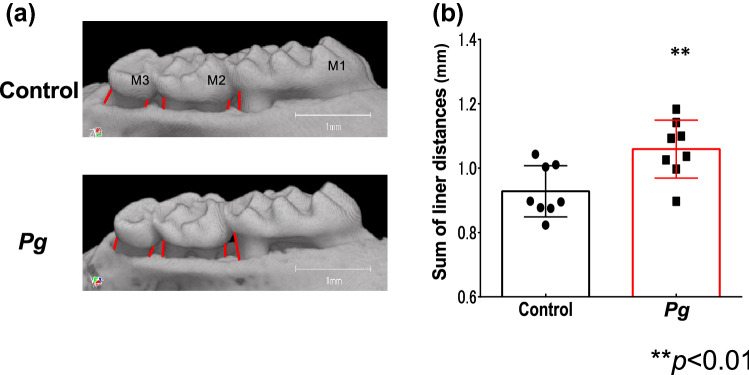
Effects of oral *Porphyromonas gingivalis* (*Pg*)*-*administration on alveolar bone loss in *db*/*db* mice (**a**) Buccal-side maxillary alveolar bone loss (ABL, colored lines), measured from the cemento-enamel junction (CEJ) to alveolar bone crest (ABC) at five points: (1) distobuccal regions for first maxillary molar (M1); (2) mesiobuccal (3) distobuccal regions for second maxillary molar (M2); (4) mesiobuccal and (5) distobuccal regions for third maxillary molar (M3), in *db*/*db* mice treated with *Pg* or CMC control for 30 d. (**b**) Comparison of the sum of the five CEJ-ABC linear distances. **P < 0.01; *n* = 8. Data are shown as mean ± SEM.

To assess the severity of the periodontitis, bone loss on the buccal side of the maxillary alveolar bone was measured at five points using μCT image analysis. *Pg*-treated mice exhibited statistically significant (*P* < 0.01) alveolar bone resorption, compared to that in control mice (Fig. 1a,b).

### Detection of *Pg*-specific peptides and changes in the microbiome profile of fecal specimens

The metaproteome profiling of the fecal samples was analyzed using the LC–MS/MS-based shotgun proteomics. Approximately 350,000 MS/MS spectra per sample were obtained and were screened against the UniProt database of all putative proteins in the mouse gut metagenome^21^, the murine UniProt proteome database, and against the proteomes of the food items, and that of *Pg*. The number of peptides identified in the proteome analysis did not differ substantially between the *Pg-* and CMC-treated groups (Table 4). In total, 16,974 unique peptides were identified. Among them, 5576 taxon-specific peptides were matched to 14 phyla of microbes, 2451 peptides to 58 families, and 1626 peptides to 111 genera (Supplementary Table S1).

**Table 4:** Number of peptides detected during the proteome profiling of the feces of the *Porphyromonas gingivalis* (*Pg*)- and carboxymethyl cellulose-treated *db*/*db* mice.

Category	1st administration		10th administration	
	*Pg*	Control	*Pg*	Control
All	7000	6641	9064	8619
Mouse	844	821	1408	1276
Bacteria	5467	5061	6601	6324
Food	689	759	1019	1019

There was no statistical difference in the number of peptides detected, between the *Pg*- and CMC control-treated groups.

**Figure 2:**
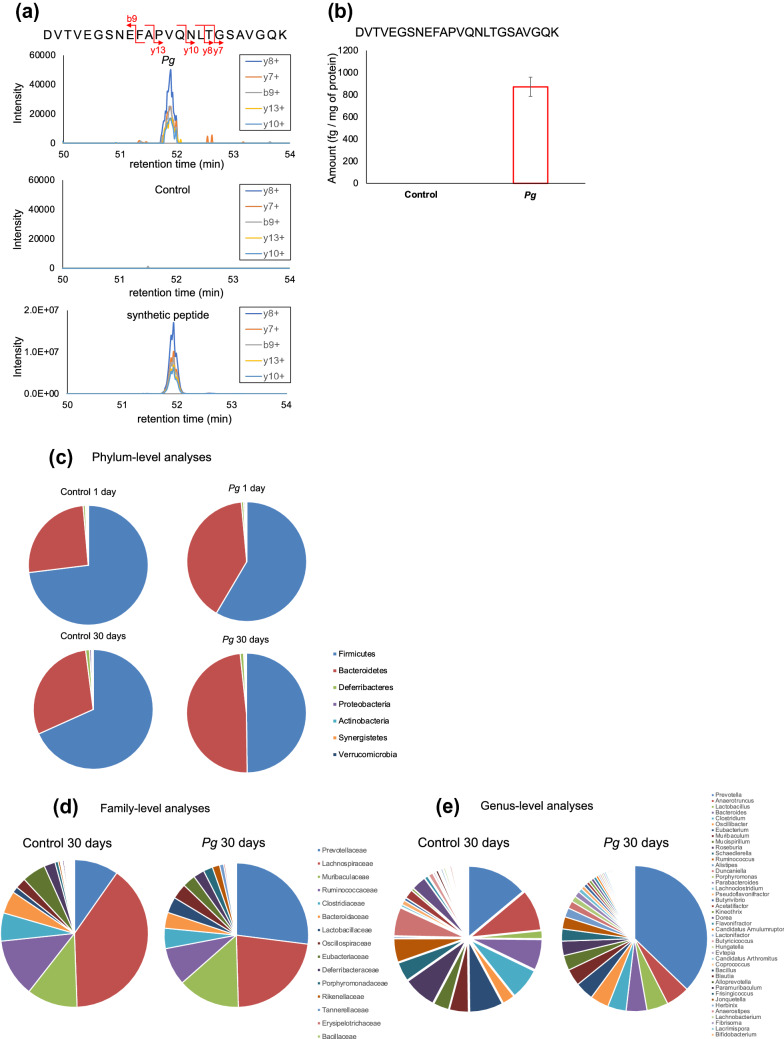
Parallel reaction monitoring analysis of the distinct peptides derived from *Porphyromonas gingivalis* (*Pg*)*.* (**a**) Chromatogram of the fragment ions for the *Pg*-unique peptide, DVTVEGSNEFAPVQNLTGSAVGQK and its five different amino-acid fragments in *Pg*- or control-treated mouse feces, and that of the synthetic peptide used as the internal standard. (**b**) Detection of this peptide in *Pg*-treated, but not CMC control-treated, mice, using a synthetic peptide as internal standard. Sample preparation was performed in triplicate from the pooled feces; peptides were quantified based on the median peak area ratio of each fragment ion. (**c**) Phylum-, (**d**) family-, and (**e**) genus-level distributions of the fecal microbiome. Taxonomic assignments of the peptides identified via metaproteome analysis of fecal samples were performed using Unipept. The distributions were profiled based on the number of taxa-specific peptides. ‘Control 1 day’: CMC control-treated, 1 d after the first injection; ‘Control 30 days’: CMC control-treated, 30 d after the tenth injection; ‘*Pg* 1 day’: *Pg*-treated, 1 d after the first injection: ‘*Pg* 30 days’: *Pg*-treated, 30 d after the tenth injection.

Six distinct peptides derived from *Pg* were specifically identified in *Pg*-treated *db*/*db* mice (Table 5). The specific detection of the *Pg*-derived peptides in *Pg*-treated mice were quantitatively assessed using parallel reaction monitoring (PRM) analysis with a synthetic peptide as an internal standard. *Pg*-specific peptides were detected in the fecal specimens from *Pg*-treated, but not in that from the CMC (control)-treated, mice (Fig. 2a,b and Supplementary Figure S1).

**Table 5:** Distinct peptides of *Porphyromonas gingivalis* (*Pg*) derived from the feces of *Pg*-treated and CMC-treated *db*/*db* mice using proteome analysis.

Peptide sequence	Corresponding protein
DVTVEGSNEFAPVQNLTGSAVGQK	Hemagglutinin A
ECVNVTVDPVQFNPVQNLTGSAVGQK	Hemagglutinin A
NDSNTSDYSIIFNTLQK	DNA-directed RNA polymerase subunit beta
LQFTGFDIYGFPQGSK	Outer membrane protein 40
VAEDIASPVTANAIQQFVK	Gingipain R1
VLVDNYPLIDVTTAK	Receptor antigen B

The phylum-level microbial composition of the fecal microbiome was profiled based on the metaproteome analysis. Bacteroidetes and Firmicutes dominated the gut microbiota in both *Pg*-treated and control mice (Fig. 2c). After the 30 day treatment period, the Bacteroidetes population (as a proportion of all bacteria present) was larger; while, the Firmicutes population was smaller in the *Pg*-treated mice, compared to that in the control group (Firmicutes/Bacteroides: 68%/30% in the *Pg*-treated group and 50%/49% in the control group, respectively). In addition, the changes in the microbiome were larger following the 10th *Pg*-treatment period, compared to that after a single treatment of *Pg* (Fig. 2c). At the family level, *Prevotellaceae* constituted a higher proportion in the *Pg*-treated group (27%), compared to that in the control (10%) (Fig. 2d). *Prevotella*, the most abundant genus in the fecal samples, was present at a higher proportion in the *Pg*-treated group (37%), compared to that in the control (14%) (Fig. 2e).

### Changes in the intestinal metabolites following *Pg* treatment

**Figure 3:**
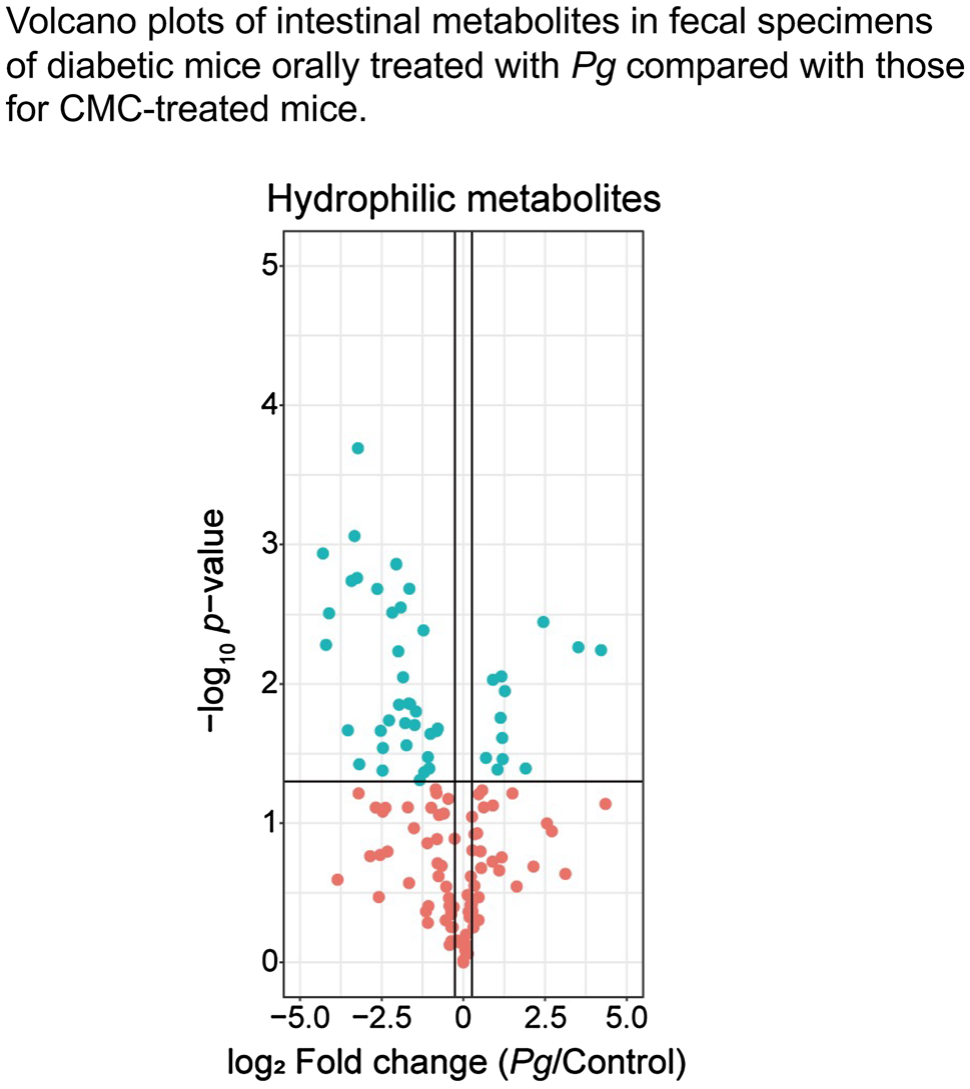
Volcano plot of fold change in the levels of intestinal hydrophilic metabolites in fecal specimens of *db*/*db* diabetic mice orally treated with *Porphyromonas gingivalis* (*Pg*) or CMC (control). Blue points represent a significant increase or decrease.

Metabolome analysis of mixed samples of small intestinal tissues and fecal materials showed marked differences in the intestinal metabolites between *Pg*- and CMC-treated mice. The volcano plots were used to compare the metabolite levels in the fecal specimens of *Pg-* and CMC-treated mice. Following *Pg* treatment, the levels of 12 hydrophilic metabolites were significantly elevated; while, that of 35 other metabolites were significantly reduced (Fig. 3). Many end metabolites, such as lactate, phosphoric acid, 3-hydroxybutyric acid, 3-hydroxyisobutyric acid, a valine metabolite, and *O*-phosphoethanolamine, a metabolite of sphingosine-1-phosphate, were significantly higher in the *Pg*-treated mice, compared to that in the control mice (Table 6). In contrast, the levels of many amino acids and polyamines were significantly lower in the *Pg* treated mice, compared to that in the control mice (Table 6).

**Table 6:** Intestinal metabolites with significantly different expression in the *Porphyromonas gingivalis* (*Pg*)- and carboxymethyl cellulose-treated *db*/*db* mice.

Downregulation			Upregulation		
	*Pg*/Control	*P* value		*Pg*/Control	*P* value
Spermine	0.010	0.00087	Fructose 1-phosphate	2.7	0.01357
Tyramine	0.050	0.00520	3-Hydroxybutyric acid	2.3	0.03464
Cytosine	0.060	0.00310	3-Hydroxyisobutyric acid	2.3	0.02438
Tryptophan	0.090	0.00180	Fructose 1-phosphate	2.2	0.01465
Histidine	0.090	0.02100	O-Phosphoethanolamine	1.9	0.00931
Lysine	0.10	0.00170	Phosphoric acid	1.6	0.03399
Tyrosine	0.11	0.00020	Lactic acid	1.5	0.01121
Ornithine	0.14	0.00560			
Arginine	0.16	0.00210			
Lactose-meto	0.17	0.02200			
Asparagine	0.18	0.02900			
Lactose	0.18	0.04200			
Phenylalanine	0.22	0.00310			
*N*-Acetyl glutamine	0.24	0.00138			
Gluconic acid	0.25	0.00580			
Galacturonic acid	0.26	0.01400			
Arabitol	0.27	0.00280			
Dihydroxyacetone	0.28	0.00890			
Allantoin	0.29	0.01900			
Pyruvic acid	0.30	0.02800			
Octopamine	0.32	0.00210			
Leucine	0.32	0.01400			
Methionine	0.36	0.02000			
Isoleucine	0.37	0.01600			
Glutamine	0.40	0.04900			
Isoleucine	0.42	0.00037			
Pantothenic acid	0.42	0.00330			
Arabinose-meto	0.43	0.00410			
Glutamic acid	0.44	0.04300			
Valine	0.47	0.03300			
Fructose-meto	0.47	0.03900			
Fructose	0.49	0.04000			
Ornithine	0.50	0.02300			
5-Oxoproline	0.57	0.02200			
Cysteine	0.58	0.02100			

Intestinal metabolite levels were measured using metabolome analysis of mixed intestinal tissue and fecal material specimens. Data are expressed as mean ± standard error of mean; *n* = 3.

### Changes in the expression of rate-limiting enzymes and the levels of glucose metabolites in the liver of *Pg*-treated diabetic mice

**Figure 4:**
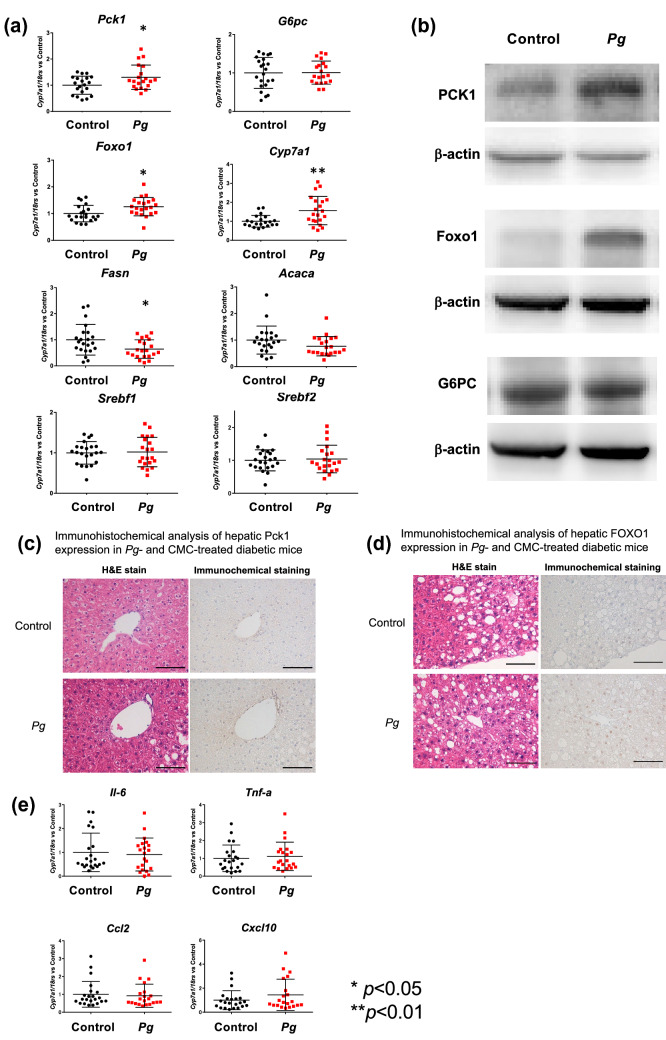
Effects of oral *Porphyromonas gingivalis* (*Pg*) administration on mRNA and protein expression in the liver of *db*/*db* mice. (**a**) mRNA expression of *Pck1*, *G6pc*, *Foxo1*, *Cyp7a1*, *Fasn*, *Acaca*, *Cpt1*, *Srebf1*, and *Srebf2*. mRNA expression was determined using quantitative RT-PCR and was normalized against the expression of *18S rRNA* mRNA. Target gene expression in *Pg*-treated mice was normalized against the target gene expression in CMC-treated mice, which is considered as 1. Treatment or control group variability was calculated as the ratio of [each amount/the mean amount in the control] in every chart. *n* = 20–21, **P < 0.01, *P < 0.05 versus control. (**b**) Western blot analysis of PCK1, G6PC, and FOXO1 in liver tissues from *db*/*db* mice treated with *Pg* or CMC for 30 days. β-actin was used as the loading control. The bar graphs on the right show densitometric quantification of the amounts of PCK1, G6PC, and FOXO1, relative to that in the control. *n* = 4; *P < 0.05 versus control. (**c**, **d**) PCK1 (**c**) and FOXO1 (**d**) detection in liver tissues of *Pg*- or control-treated mice. Left column: Paraffin-embedded sections stained with hematoxylin and eosin (H&E). Right column: Immunohistochemical detection. Scale bars: 100 μm. (**e**) Comparison of the relative gene expression of proinflammatory cytokines (*Il-6*, *Tnf-α*, *Ccl2*, and *Cxcl10*) in the liver tissues of *Pg*- and control-treated *db*/*db* mice. The data were normalized and analyzed as described for (**a**). *n* = 20–21. Pck1, Phosphoenolpyruvate carboxykinase 1; G6pc, Glucose-6-phosphatase; Foxo1, Forkhead box protein O1; Cpt1c, Carnitine palmitoyltransferase 1c; Fasn, Fatty acid synthase; Acaca, Acetyl-Coenzyme A carboxylase alpha; Srebf1, Sterol regulatory element-binding transcription factor 1; Srebf2, Sterol regulatory element-binding transcription factor 2; Il-6, Interleukin 6; Tnf-α, Tumor necrosis factor-α; Ccl2, Chemokine (C–C motif) ligand 2**;** Cxcl10, C-X-C motif chemokine ligand 10.

Enhanced fasting hyperglycemia is regulated by hepatic gluconeogenesis in poorly controlled diabetes. To determine whether the expression of hepatic genes related to gluconeogenesis were upregulated in *Pg*-treated mice, the expression of phosphoenolpyruvate carboxykinase (*Pck1*) and Glucose 6 phosphatase (*G6pc*) was evaluated. *Pck1* mRNA expression was significantly higher in *Pg*-treated mice, compared to that in the control mice (*P* < 0.05); however, *G6pc* mRNA expression did not differ significantly between the *Pg*-treated and CMC-treated mice (Fig. 4a). Expression of Forkhead box protein O1 (*Foxo1*), a transcription factor in hepatocytes that promotes gluconeogenesis by activating *Pck1* and *G6pc* expression, was also significantly higher in the *Pg*-treated mice, compared to that in the control. *Cytochrome P450 A1* (*Cyp 7a1*), a rate-limiting enzyme in bile acid biosynthesis, was significantly (*P* < 0.01) upregulated in the livers of *Pg*-treated mice, compared to that in the control. Western blot analysis revealed higher levels of PCK1 and FOXO1 protein expression in *Pg*-treated mice (*P* < 0.05), compared to that in the control (Fig. 4b). Immunohistochemical analyses indicated upregulated PCK1 and FOXO1 expression in the livers of *Pg*-treated mice, compared to that in the control (Fig. 4c,d). However, the expression of fatty acid synthase (*Fasn*) was significantly downregulated (*P* < 0.05), while that of acetyl-CoA carboxylase A (*Acaca*) was lower in the livers of *Pg*-treated mice, compared to that in the control. The expression of hepatic lipogenesis-related genes, *Srebf1* and *Srebf2*, did not differ significantly between the *Pg*-treated and control mice (Fig. 4a). mRNA expression of Il-6, Tnf-α, Ccl2, and Cxcl10 were not significantly different between the *Pg*-treated and control mice (Fig. 4e).

**Figure 5:**
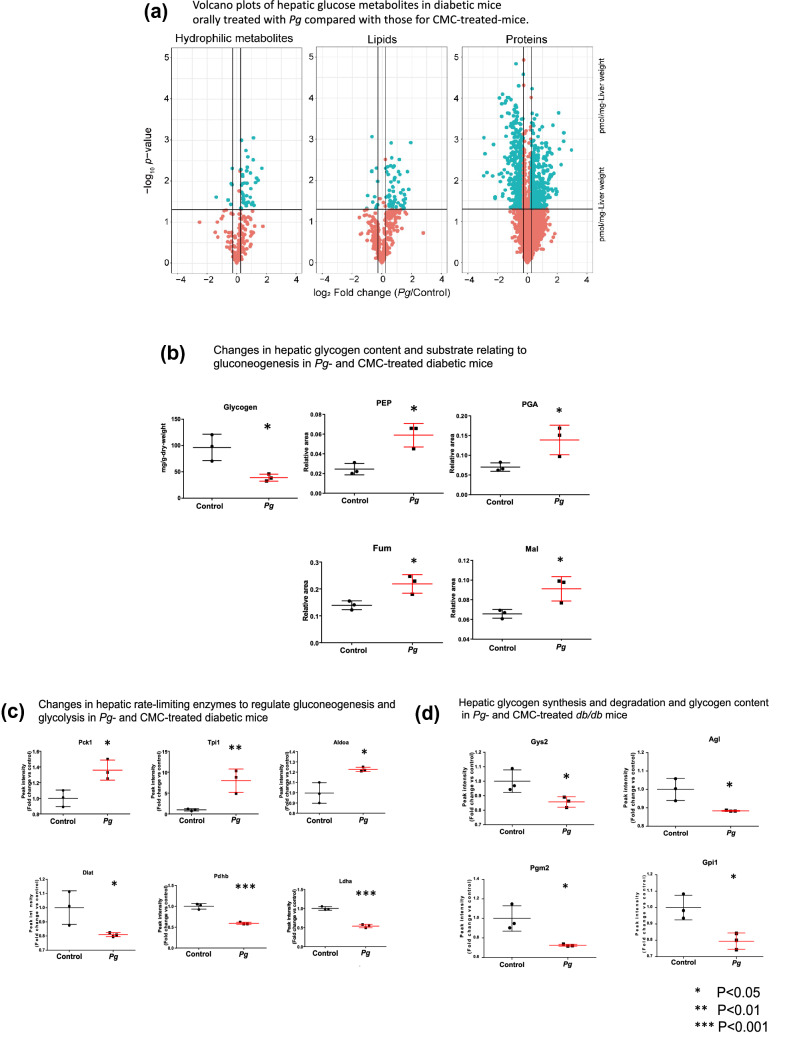
Proteome and metabolome profiles of the livers of *db*/*db* mice treated with *Porphyromonas gingivalis* (*Pg*) or CMC for 30 days, obtained using nano-LC/MS/MS, in triplicate. (**a**) Volcano plots of fold change in the levels of hydrophilic metabolites, lipids, and proteins, between *Pg-* and CMC-treated mice. Blue points represent significant increase or decrease (the relevant compounds are listed in Supplementary Tables S3 and S4–7). (**b**) The levels of glucose metabolites (PEP, PGA, FUM, and MAL) were significantly higher (*P* < 0.05), while the glycogen content was significantly lower (*P* < 0.05), in *Pg-*treated mice, compared to that in CMC-treated mice. (**c**) Comparative proteome analysis of the levels of glycolysis/gluconeogenesis-related enzymes in *Pg*- and CMC-treated mice. (**d**) Comparative proteome analysis of enzyme synthesis and degradation during glycogen and G6P metabolism via the glycolytic pathway in *Pg-* and CMC-treated mice. PCK1, ALDOA, and TPI1 were significantly upregulated; while, Pgm2, DLAT, GPI1, LDHA, PDH, Gys2, AGL, and Gpi 1 were significantly downregulated (**c, d**), in the *Pg*-treated mice, compared to that in the CMC-treated mice. Panels (**b**–**d**) Data normalized and statistically analyzed as for Fig. 4a. *n* = 3, *P < 0.05, **P < 0.01 and ***P < 0.001, versus control.

**Figure 6:**
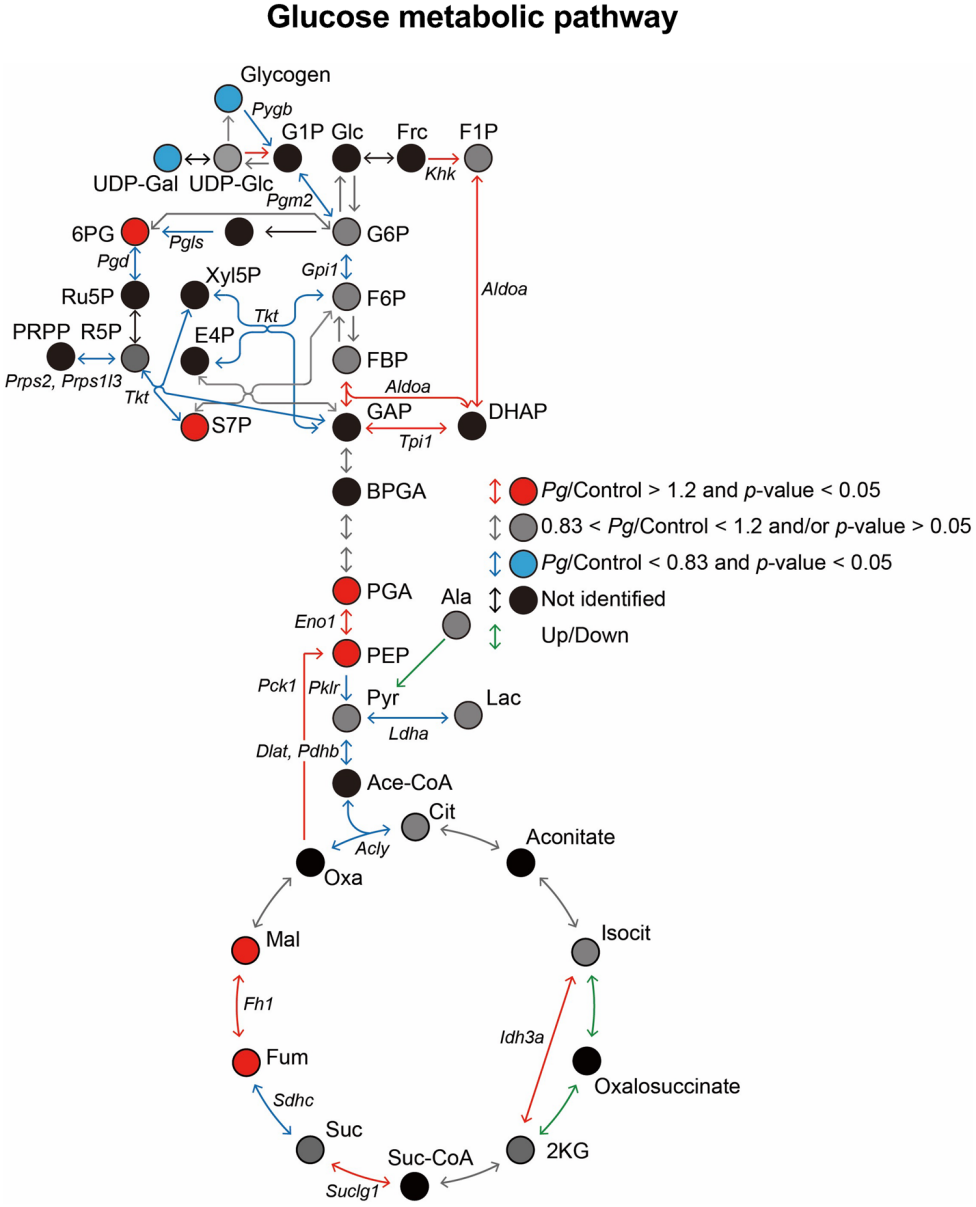
Schematic presentation of the levels of hepatic glucose metabolites and the expression of rate-limiting enzymes of glucose metabolism in *Pg*- and CMC-treated diabetic mice. Fold changes in the glucose metabolites and enzymes involved in intrahepatic glucose metabolism in the *Pg*-treated mice, relative to that in the control. Red circles and lines: significant increases. Blue circles and lines: significant reductions. Metabolites that varied substantially and significantly between the *Pg*-treated mice and the control were identified using these criteria: log_2_ fold change >|0.26| and *P* < 0.05, and *P* < 0.01. 2 KG, 2-Ketoglutaric acid; 6PG, 6-Phosphogluconic acid; Ace CoA, Acetyl-CoA; BPGA, 1,3-Bisphosphoglycerate; Cit, Citric acid; DHAP, Dihydroxyacetone phosphate; F1P, d-Fructose 1-phosphate; F6P, d-Fructose 6-phosphate; Frc, d-Fructose; G1P, d-Glucose 1-phosphate; G6P, d-Glucose 6-phosphate; GAP, Glyceraldehyde 3-phosphate; Glc, d-Glucose; Isocit, Isocitric acid; Lac, Lactic acid; MAL, Malic acid; Oxa, Oxaloacetic acid; PEP, Phosphoenolpyruvic acid; PGA, 2-Phospho-d-glyceric acid/3-Phospho-d-glyceric acid; Pyr, Pyruvic acid; Suc, Succinic acid; Suc CoA, Succinyl-CoA; UDP-Gal, UDP-alpha-d-galactose; UDP-Glc, Uridine 5′-diphosphate glucose; AGL, Amylo-1,6-glucosidase, 4-alpha-glucanotransferase; Aldoa, Fructose-bisphosphate aldolase A; Dlat, Dihydrolipoyllysine-residue acetyltransferase component of pyruvate dehydrogenase complex, mitochondrial; Pdhb, Pyruvate dehydrogenase E1 component subunit beta; Fh1, Fumarate hydratase-1; Gpi1, Glucose-6-phosphate isomerase 1; Gys2: Glycogen [starch] synthase 2; Ldha, L-lactate dehydrogenase; Pck1, Phosphoenolpyruvate carboxykinase; Pgm2, Phosphoglucomutase-2; Idh3a, Isocitrate dehydrogenase [NAD] subunit alpha, mitochondrial; Suclg1, Succinyl-CoA ligase [GDP-forming] subunit alpha, mitochondrial; Sdhc, Succinate dehydrogenase (ubiquinone) cytochrome b560 subunit; Acly, ATP citrate (pro-S)-lyase; Tpi1, Triosephosphate isomerase 1.

To understand the enhanced fasting hyperglycemia in diabetic mice following oral *Pg*-administration, the levels of enzymes, glucose metabolites, and lipid metabolites in the liver were quantified through proteomic and metabolomic analyses. There was significant differential expression level between *Pg*-treated and the control groups, such as a 1.2-fold increase, or more than a 0.83-fold reduction, in *Pg*-treated mice relative to levels in the control (*P* < 0.05). Volcano plots of the hepatic glucose metabolites (Fig. 5a) indicated that the levels of 396 proteins, 42 hydrophilic metabolites, and 62 lipids were elevated significantly following *Pg*-treatment. The levels of 444 proteins, 6 hydrophilic metabolites, and 12 lipids decreased following the *Pg*-treatment (Fig. 5a). Comparative metabolomic analysis revealed that *Pg-*administration significantly reduced the glycogen storage in the liver and increased the levels of metabolites related to gluconeogenesis and the tricarboxylic acid cycle (TCA) cycle, such as phosphoenolpyruvic acid (PEP), phosphoenolpyruvic acid (PGA), fumaric acid (FUM), and malic acid (MAL), in the liver (*P* < 0.05) (Fig. 5b). The levels of glycolysis/gluconeogenesis-related proteins such as PCK1, Tpi1, and Aldoa, were significantly (*P* < 0.05) higher (Fig. 5c); while, that of Diat, Pdhb, and Ldha were significantly lower (*P* < 0.05) in the treated mice, compared to that in the control (Fig. 5d). Comparative proteome analysis revealed that the levels of the enzymes involved in glycogen synthesis and degradation, and in the glucose 6-phosphate (G6P) metabolism via the glycolytic pathway (such as Gys2, AGL, Pgm2, and Gpi1) were significantly lower in the treated mice, compared to that in the control. Changes in the levels of hepatic glucose metabolites and the expression of rate-limiting enzymes of glucose metabolism, which were comparable to enhanced gluconeogenesis in the *Pg*-treated diabetic mice as compared with the CMC-treated mice (Fig. 6). There were no significant differences in the levels of fatty acids, glycerides, cholesterol, cholesterol esters, phospholipids, and sphingolipids, between the *Pg*-treatment and the control groups (Supplementary Fig. S2).

## Discussion

Specific *Pg*-derived peptides were present in the fecal specimens from *db*/*db* diabetic mice after 30 days following oral *Pg*-administration. *Pg* treatment significantly altered the gut microbiota composition at the phylum, family, and genus levels. The levels of various intestinal metabolites were also altered in the mixed intestinal tissue and feces samples from *Pg*-treated mice. These changes were associated with aggravated reabsorption of maxillary alveolar bone and with both fasting and postprandial hyperglycemia. Proteomic and metabolomic analyses revealed that these metabolic changes were associated with the differential expression of the rate-limiting enzymes of glucose metabolism in the liver and with the levels of intrahepatic glucose metabolites, but not with the changes in whole body insulin resistance and the expression of hepatic proinflammatory cytokines.

Our findings confirm the hypothesis that oral bacteria mixed with saliva and food pass through the stomach and reach the intestinal tract. A previous metagenome analysis^17^ reported following oral *Pg*-treatment, the proportions of *Firmicutes* and *Bacteroides* was 55.4% and 38.7%, respectively, in *Pg*-treated mice, and 72.8% and 17.0%, in the control mice, respectively. In the present study, we confirmed these findings through proteomic analysis.

*Prevotellaceae* and *Prevotella* populations showed the largest increase in abundance in *Pg*-treated mice, compared to that in the CMC-treated mice (Fig. 2d,e). There are close links between the proportion of *Prevotella* and oral and gastrointestinal tract diseases^22,23^. Therefore, we intend to study the response of *Prevotella* species in the intestinal microbiota to oral administration of periodontal bacteria. The oral administration of a periodontal pathogenic bacterium, *Aggregatibacter actinomycetemcomitans*, alters the gut microbiota composition in a non-diabetic mouse model^16^, with enhanced hepatic fat deposition. This differs from the present results. Furthermore, gut dysbiosis induced by periodontal pathogens is also associated with other biological effects, such as increased intestinal permeability to low molecular weight metabolites produced by invading bacteria. These metabolites are delivered to the liver, where they could impair glucose tolerance and enhance insulin resistance, while activating the expression of proinflammatory molecules^15,16^. However, several studies have also indicated that the changes in the levels of specific beneficial metabolites improve whole-body glucose metabolism, the stabilization of intestinal barrier function^24,25^, or help control obesity^26^. In this study, we observed remarkable changes in the levels of various intestinal metabolites following *Pg* treatment, without difference in expression of proinflammatory cytokines in the liver between the two groups.

The liver is crucial for maintaining normal glucose homeostasis, in regulating glycogen synthesis and degradation, glycolysis, and gluconeogenesis, depending on the fasting and postprandial states. In type 2 diabetes with poor glycemic control, the hepatic glucose output is regulated by gluconeogenesis^18,19^, through changes in the levels of insulin, insulin counter-regulatory hormones, and the supply of gluconeogenic substrates^19,27,28^. Oral administration of *Pg* upregulated the expression of hepatic gluconeogenesis-related enzymes at the mRNA and protein levels in *db*/*db* mice. Oral administration of periodontal pathogens impairs both glucose tolerance and insulin sensitivity in non-diabetic mice and in streptozotocin-induced diabetic mice^15,16,20^; this could be attributed to the increased expression of hepatic proinflammatory cytokines^15,20^. However, in the present study, *Pg* treatment did not alter the expression of proinflammatory cytokines in *db*/*db* obese type 2 model mice treated with *Pg*, compared to the corresponding expression levels in CMC-treated *db*/*db* mice (Fig. 4e). Obese type 2 diabetes model mice extensively exhibit insulin resistance and hyperglycemia^29,30^. Based on the insulin tolerance test (ITT), *db*/*db* diabetic mice in both *Pg-* and CMC-treated groups were equally insulin resistant^31,32^, because of an impaired reduction in the plasma glucose levels at 30–60 min after insulin loading. These results are in line with the lack of further changes in the expression of proinflammatory cytokines in the liver of *db*/*db* mic treated with *Pg*. There was an increase in the fasting hyperglycemia without changes in the fasting serum insulin levels, indicating that impaired fasting insulin secretion could be a cause for fasting hyperglycemia. The exact molecular mechanisms that induce the progression of fasting hyperglycemia in *db*/*db* mice treated with *Pg*, warrants further studies. Evaluating the role of various gut factors in triggering hepatic gene expression, using portal vein samples from *db*/*db* mice orally treated with *Pg*, in comparison to that in CMC-treated *db*/*db* mice, is required. *Pg* administration induces fatty liver and increases the hepatic triglyceride levels in a non-diabetic mouse model^15^. However, our results indicated that orally administered *Pg* did not modify the triglyceride levels in either the blood or liver, compared to that in the CMC-treated diabetic mice. In addition, it did not increase the expressions of fatty acid biosynthesis-related enzymes, such as Fasn, Acaca, Srebf1, and Srebf2.

This study is focused on the upregulation of hepatic gluconeogenesis following oral *Pg* administration in diabetic mice. FOXO1 is the most direct transcriptional regulator of gluconeogenesis^33^. We observed consistently upregulated *FOXO1* and *PCK1* expression in *Pg*-treated diabetic mice; however, there were no significant changes in the expression of *G6pc*. The activation of *FOXO1* and *PCK1* could influence gluconeogenesis; however, it remains unclear whether the dysregulation of the *FOXO1* is the main contributor to the increased rates of gluconeogenesis in type 2 diabetes^28,34^. In addition, the extrahepatic supply of gluconeogenic precursor substrates such as glycerol, lactate, and alanine from fat and muscle cells could be necessary in vivo. In the present study, we used the same liver samples for metabolomic and proteomic analyses. *Pg* treatment reduced the levels of stored glycogen; this is consistent with the results from previous studies, in patients with type 2 diabetes^35^. Glycerol released from the fat cells is converted to dihydroxyacetone phosphate (DHAP) and then glyceraldehyde 3-phosphate (GAP) via Tpi1 in the liver (Fig. 6). These metabolites are essential for gluconeogenesis, for the conversion of GAP to fructose-1,6-bisphosphate (FBP) and DHAP to fructose 1-phosphate (F1P) via aldolase (*Aldoa*). Both *Aldoa* and *Tpi1* were upregulated in *Pg*-treated mice. Alanine released from skeletal muscle cells is converted to pyruvate via alanine aminotransferase (*ALT*), and lactate is converted to pyruvate via lactate dehydrogenase (*Ldha*) in the liver. Pyruvate is then converted to oxaloacetate via pyruvate carboxylase. Oxaloacetate is then converted to PEP via PCK1. Our western blotting, proteomic analyses, and real time PCR analysis revealed increased levels of PCK1 protein and mRNA expression following *Pg* treatment. However, the expression of lactate dehydrogenase and pyruvate dehydrogenase B was downregulated, indicating a limited pyruvate flow to the TCA cycle, and an increased pyruvate flow to the gluconeogenesis pathway. In addition, the levels of both FUM and MAL were upregulated following *Pg*-treatment; it is possible that these metabolites promote hepatic gluconeogenesis by supplying oxaloacetate. Therefore, the altered levels of hepatic glucose metabolites and rate-limiting glucose-metabolism enzymes following *Pg*-treatment are consistent with enhanced gluconeogenesis in *Pg*-treated *db*/*db* mice. In addition, consistent with previous reports^35^, *Pg* treatment reduced glycogen storage in the liver of *db*/*db* mice, indicating that the treatment reduced glucose incorporation into glycogen. However, such changes in the levels of hepatic glucose metabolites could be induced by increased plasma insulin-counter regulatory hormone levels^29,36^, which were not extensively measured in the present study. *FOXO1* mRNA expression is increased in the cultured gingival epithelial cells treated with *Pg*^37^. The enhanced FOXO1 expression could modulate multiple keratinocyte functions. Understanding the underlying molecular mechanisms of FOXO1 expression is valuable to be tested in the hepatocytes through the specific *Pg*-derived peptides and the specific *Pg*-induced changes in intestinal metabolites and bacterial flora as one of the future projects. Specifically, it is necessary to evaluate the role of various gut factors in triggering hepatic gene expression using the specifically identified factors in portal vein samples from *Pg*-treated *db*/*db* mice compared with that in CMC-treated *db*/*db* mice.

The findings of this study might not be sufficient to explain the relationship between the change of gut microflora and exacerbation of gluconeogenesis. The altered gene expression in the liver after *Pg* administration could be because of *Pg* or its components being associated with the alternation. Unfortunately, however, it was very difficult to detect *Pg* or its components in the liver of this mouse model. Thus, we alternatively stimulated HepG2 cells (human hepatoma cell line) with *Pg-*derived LPS or human recombinant IL-1β as positive control and examined mRNA expressions of CCL2, CXCL10, and FOXO1 using real time PCR. Interestingly, *Pg-*derived LPS increased the mRNA expression of IL-6, CCL2, CXCL10, and FOXO1 (Supplementary Fig. S3). This suggests that *Pg* or its components may upregulate the expression of both FOXO1 and inflammatory cytokines in the liver. In contrast, the expression of Foxo1 but not Il-6, Ccl2 and Cxcl10 was increased in the liver (Fig. 4e). We clearly demonstrated that oral *Pg* treatment profoundly alters the gut microbiome profiles at the phylum, family, and genus levels. Particularly, *Prevotella* showed the largest increase in abundance following *Pg* treatment. Moreover, there were profound metabolite changes in the intestinal and fecal samples. Taken together, we speculate that specific metabolites generated by the changes in the intestinal microbiota may affect the expression of Foxo1 and the enhancement of gluconeogenesis without affecting inflammatory cytokine levels in the liver. Identification of the gut factors or specific metabolites triggering the expression of hepatic genes could be an important aspect to be investigated in future studies using the portal vein samples from the *Pg-*administered *db*/*db* mice*.*

In conclusion, we identified *Pg*-specific peptides in fecal specimens of obese type 2 diabetes model mice orally administered with *Pg* for 30 days. Proteomic analysis revealed that the presence of *Pg* in the intestine significantly altered the gut microbiome profile at the phylum, family, and genus levels. *Pg* treatment significantly altered the levels of intestinal end metabolites, several amino acids, and polyamines. *Pg* treatment aggravated both fasting and postprandial glucose levels, and increased the levels of gluconeogenesis-related metabolites and enzymes. However, there was no increase in the expression of proinflammatory cytokines in the liver and insulin resistance in the *Pg* treated *db*/*db* mice, which are typical characteristics in obese type 2 diabetes model mice.

## Methods

### Animals

Forty-one male *C57BLKS*/*Jlar*- + *Lepr*/*db* + *Lepr*/*db* (*db⁄db*) mice aged 6 weeks were purchased from Japan SLC, Inc. (Shizuoka, Japan). They were maintained under controlled temperature (23 ± 2 °C) and light–dark cycle with free access to food and water, and fed a regular chow diet (5.1% fat, 55.3% carbohydrate, 23.1% protein; MF Oriental Yeast Co., Ltd., Tokyo, Japan). After acclimatization for a week, the mice were randomly assigned to *Pg-*treated (*n* = 20) and CMC-treated (*n* = 21) groups. The bacterial load administered in the mouse periodontitis model was based on Baker et al.^38^. *Pg* and CMC were administered orally through a plastic tube, with 10^9^ CFU *Pg* mixed with 4% CMC (for the *Pg*-treatment), or only CMC (for the control), every 3 days for 30 days. The experiment was divided into four administrating sessions with 4–6 animals in each group. The food intake in *db*/*db* mice was measured for 6–11 consecutive weeks. The animals and the amount of food in the cage were weighed once a week. After anesthetization using mixed anesthesia (Domitor, 0.75 mg/kg body weight; Midazolam, 4 mg/kg; and Butorphanol Tartrate, 5 mg/kg), blood was collected from inferior vena cava and liver samples were excised and harvested for the following experiments. Maxillae were removed from euthanized *db⁄db* mice and fixed using 4% paraformaldehyde for 48 h.

The oral glucose tolerance test was performed following overnight (10 h) fasting, 21 days after the initial *Pg* treatment. Fasting glucose levels were measured, and mice were orally administered with 2 g glucose/kg body weight. The intraperitoneal insulin tolerance test was conducted with intraperitoneal insulin injections (5 units/kg body weight), 26 days after the initial *Pg* treatment. Blood glucose levels were measured at 0, 30, 60, and 120 min after insulin administration.

To determine insulin levels, blood samples were collected from the inferior vena cava of anesthetized mice. Serum insulin levels were determined using the insulin ELISA kit (FujiFilm Wako Shibayagi Corporation, Gunma, Japan), following the manufacturer’s instructions.

All animal experiments were performed according to the protocols approved by the institutional animal care and use committees of Osaka University Graduate School of Dentistry (permit number: 27–022-0). In addition, all methods were performed in compliance with the ARRIVE guidelines.

### Bacterial culture

The *Pg* strain (ATCC33277) was obtained from the American Type Culture Collection (ATCC, Manassas, VA) and grown at 37 °C for 24 h in an anaerobic box chamber (Mitsubishi Gas Chemical Company, Inc. Tokyo, Japan) with AnaeroPack-Anaero anaerobic gas generator (Mitsubishi Gas Chemical Company, Inc.) in Gifu anaerobic medium supplemented with 5 mg/mL yeast extract, 5 μg/mL hemin, and 0.2 μg/mL vitamin K1.

### Quantification of alveolar bone resorption

Morphometric analysis of the buccal alveolar bone resorption was performed using an R_mCT2 3D micro X-ray computed tomography system designed for use with scanned images of laboratory animals (Rigaku, Tokyo, Japan). An examiner blinded to the experimental groups measured the linear distances of the cemento-enamel junction (CEJ) from the alveolar bone crest (ABC) using the 3D image analysis software TRI/3D-BON (RATOC System Engineering Co., Ltd., Tokyo, Japan). Buccal-side maxillary alveolar bone loss (ABL) was measured from the cemento-enamel junction (CEJ) to alveolar bone crest (ABC) at five points: (1) distobuccal regions for first maxillary molar (M1); (2) mesiobuccal and (3) distobuccal regions for second maxillary molar (M2); and (4) mesiobuccal and (5) distobuccal regions for third maxillary molar (M3), after 30 days following the treatment with *Pg* or CMC in *db*/*db* mice. Distance between the CEJ and the ABL was measured at five sites in the apical direction using WinROOF software version 7.4 (https://www.mitani-visual.jp/products/#image_analys_ismeasurement) (Mitani Corporation, Fukui, Japan), and total value of five points on the μCT image was defined as the alveolar bone loss, were compared in *Pg* or CMC-control treated groups. Prior to the observation, the intraclass correlation for the evaluation of bone loss measurements was examined. One examiner evaluated the same teeth points on different days. The resulting intraclass correlation coefficient was 0.86.

### Real-time PCR

Total RNA from the mouse liver was extracted using a RNeasy lipid tissue mini kit (Qiagen, Venlo, Netherlands), according to the manufacturer’s instructions. cDNA was synthesized from 100 ng total RNA using a high-capacity cDNA archive kit (Applied Biosystems, Foster City, CA). PCR was performed using the ABI 7300 real-time PCR system with the Power SYBR Green PCR master mix (both from Applied Biosystems), according to the manufacturer’s protocol. To control for the variations in the amount of DNA available for PCR, target gene expression in each sample was standardized based on the expression of an endogenous control. The sequences of the primers used are provided in Supplementary Table S2.

### Protein analysis

Total proteins were extracted from the frozen liver tissues using the T-PER tissue protein extraction reagent (Thermo Fisher Scientific Inc., Waltham, MA), and used for western blotting. Immunoblotting was performed using the following primary antibodies: PCK1 (1:1000; ab28455, Abcam, Toronto, Canada), G6PC (ab83690; Abcam), FOXO1 (1:000; 2880, Cell Signaling Technology, Danvers, MA), and β-actin (A5216, Sigma‐Aldrich Co., St. Louis, MO), and incubated with anti- rabbit HRP-conjugated secondary antibody (1:10,000; NA934, GE Healthcare, Chicago, IL, USA) anti- mouse HRP (1:10,000; NA931, GE Healthcare). Immunoreactive bands were visualized using ECL (Thermo Fisher Scientific).

### Histology

Liver tissues, excised from mice after 30 days following the oral administration of *Pg* and CMC, were fixed using 4% paraformaldehyde for 48 h and embedded in paraffin. Samples were then deparaffinized, rehydrated, and washed with PBS. The tissue sections were cut at 4 μm thickness with LEICA RM2245 (Leica Microsystems, Wetzlar, Germany) and stained with hematoxylin and eosin (H&E). For immunohistochemistry, samples were embedded in paraffin, sectioned, and stained with rabbit anti-PCK1 (ab2845, 0.4 µg/mL; Abcam) and rabbit anti-FOXO1 antibodies (2880, 0.2 µg/mL; Cell Signaling Technology). Positive staining was visualized using a diaminobenzidine (DAB) in stable peroxide buffer.

### Metabolome analysis

Metabolites were extracted from the frozen small intestine or frozen liver samples using Bligh and Dyer’s method^39^. Metabolome analysis of the small intestines was performed at the Chemicals Evaluation and Research Institute (CERI, Saitama, Japan) using gas chromatography triple quadrupole mass spectrometry (GC/MS/MS) and ion-paring liquid chromatography triple quadrupole mass spectrometry (ion-pairing LC/MS/MS)^40^. The hydrophilic metabolites of the liver were analyzed using ion chromatography coupled with a high-resolution tandem mass spectrometer (IC/MS/MS) for anionic polar metabolites, such as organic acids and nucleotides^41^; and with liquid chromatography with a pentafluorophenyl propyl column coupled with a high-resolution tandem mass spectrometer (PFPP-LC/MS/MS) for cationic polar metabolites, such as amino acids^41^. The levels of free fatty acids (FAs) and cholesteryl esters (ChEs) in the liver samples were quantified using supercritical fluid chromatography with a C18 column coupled with triple quadrupole mass spectrometry (C18-SFC/MS/MS)^42^. The levels of other lipids—phosphatidylcholine (PC), phosphatidylethanolamine (PE), phosphatidylserine (PS), phosphatidylglycerol (PG), phosphatidylinositol (PI), phosphatidic acid (PA), lysophosphatidylcholine (LPC), lysophosphatidylethanolamine (LPE), monoacylglycerol (MG), diacylglycerol (DG), triacylglycerol (TG), sphingomyelin (SM), cholesterol, ceramide (Cer), and hexosylceramide (HexCer)—were quantified using SFC with a diethylamine (DEA) column coupled with triple quadrupole mass spectrometry (DEA-SFC/MS/MS)^43^. Details regarding sample preparation and the analytical conditions for the analysis of the hydrophilic and hydrophobic metabolites are provided as Supplementary Methods.

### Determination of liver glycogen content

The glycogen content in liver tissues was determined using an aqueous size-exclusion chromatographic method, as reported previously^44^.

### Proteome analysis of liver samples and gut microbiota in fecal specimens

Liver and fecal samples of the 12-week-old male *db*/*db* mice were collected 30 days after the treatment and cut into small pieces using dissection scissors. For fecal samples, 450 µL methanol was added to 10 mg feces, and 90 µL of the suspension was diluted with 450 µL methanol. Distilled water (250 µL) and 500 µL chloroform were added to the diluted suspension, followed by vertexing. After centrifugation at 4600×*g* for 5 min, both the organic and aqueous phases were removed, and pellets in the interphase were dried under vacuum. Proteins were extracted from the dried extract of the feces and the disrupted livers using the phase-transfer surfactant method^45^, with a slight modification. The extracted protein was subjected to reductive alkylation, followed by successive digestion with Lys-C endopeptidase and trypsin, as previously described^46^.

The mouse liver digests were isotopically labelled with TMT 10-plex^47^ (Thermo Fisher Scientific), according to the manufacturer’s protocol. For PRM analysis, the digests of fecal samples and that of the synthetic peptide were isotopically labelled via reductive dimethylation^48^. The digested peptides were analyzed using nano-LC/MS/MS, using an Orbitrap Fusion Lumos mass spectrometer (Thermo Fisher Scientific) in data-dependent acquisition (DDA) mode, or using a Q Exactive mass spectrometer (Thermo Fisher Scientific) in PRM mode, coupled to Ultimate3000 RSLC nano system (CTC Analytics) and the HTC-PAL autosampler (CTC). Details regarding sample preparation, the analytical conditions for nano-LC/MS/MS, and data processing, are described in the Supplementary Methods.

### Statistical analysis

All data are presented as mean ± SEM. Differences in body weight, food intake, and blood glucose levels between the *Pg* and CMC (control) groups were analyzed using one-way ANOVA with Tukey’s post hoc test. All other comparisons between the two groups were analyzed using an unpaired *t*-test. Differences were considered statistically significant at *P* < 0.05.

## Supplementary Information

Supplementary Information 1.

Supplementary Information 2.

Supplementary Information 3.

Supplementary Information 4.

Supplementary Information 5.

## Data Availability

The MS raw data and analysis files have been deposited in the ProteomeXchange Consortium (http://proteomecentral.proteomexchange.org) via the jPOST partner repository (https://jpostdb.org) with the data set identifier PXD021851. The other datasets generated during or analyzed during this study are available from the corresponding author on reasonable request.
