# Author Correction: *Porphyromonas gingivalis* induces entero-hepatic metabolic derangements with alteration of gut microbiota in a type 2 diabetes mouse model

**DOI:** 10.1038/s41598-021-99556-7

**Published:** 2021-10-04

**Authors:** Yoichiro Kashiwagi, Shunsuke Aburaya, Naoyuki Sugiyama, Yuki Narukawa, Yuta Sakamoto, Masatomo Takahashi, Hayato Uemura, Rentaro Yamashita, Shotaro Tominaga, Satoko Hayashi, Takenori Nozaki, Satoru Yamada, Yoshihiro Izumi, Atsunori Kashiwagi, Takeshi Bamba, Yasushi Ishihama, Shinya Murakami

**Affiliations:** 1 Department of Periodontology, Division of Oral Biology and Disease Control, Osaka University Graduate School of Dentistry, 1-8 Yamadaoka, Suita, Osaka 565-0871 Japan; 2 Division of Metabolomics, Medical Institute of Bioregulation, Kyushu University, Higashi-ku, Fukuoka, 812-8582 Japan; 3 Graduate School of Pharmaceutical Sciences, Kyoto University, Sakyo-ku, Kyoto, 606-8501 Japan; 4 Division for Interdisciplinary Dentistry, Osaka University Dental Hospital, 1-8 Yamadaoka, Suita, Osaka Japan; 5 Kusatsu General Hospital, Kusatsu, Shiga 525-8585 Japan

Correction to: *Scientific reports* 10.1038/s41598-021-97868-2, published online 15 September 2021

The original version of this Article erroneously included preview URL in Data availability section.

"The MS raw data and analysis files have been deposited in the ProteomeXchange Consortium (http://proteomecentral.proteomexchange.org) via the jPOST partner repository (https://jpostdb.org) with the data set identifier PXD021851 (preview URL for reviewers: https://repository.jpostdb.org/preview/13684068025f7bbef888bd0, Access key: 1026). The other datasets generated during or analyzed during this study are available from the corresponding author on reasonable request."

now reads:

"The MS raw data and analysis files have been deposited in the ProteomeXchange Consortium (http://proteomecentral.proteomexchange.org) via the jPOST partner repository (https://jpostdb.org) with the data set identifier PXD021851. The other datasets generated during or analyzed during this study are available from the corresponding author on reasonable request."

The original Article has been corrected.

