# Supplementary material for: Porphyromonas gingivalis induces entero-hepatic metabolic derangements with alteration of gut microbiota in a type 2 diabetes mouse model
Source: Sci Rep. 2021 Sep 15;11:18398. doi: 10.1038/s41598-021-97868-2 (PMC8443650; doi:10.1038/s41598-021-97868-2)
Supplement: Supplementary file 2 — Supplementary Information 2. [file 41598_2021_97868_MOESM2_ESM.docx]

**SUPPLEMENTARY INFORMATION**

***Porphyromonas gingivalis* induces entero-hepatic metabolic derangements with alteration of gut microbiota in a type 2 diabetes mouse model**

**Yoichiro Kashiwagi, Shunsuke Aburaya, Naoyuki Sugiyama, Yuki Narukawa, Yuta Sakamoto, Masatomo Takahashi, Hayato Uemura, Rentaro Yamashita, Shotaro Tominaga, Satoko Hayashi, Takenori Nozaki, Satoru Yamada, Yoshihiro Izumi, Atsunori Kashiwagi, Takeshi Bamba, Yasushi Ishihama, and Shinya Murakami**

**Supplementary Methods**

**Sample preparation for metabolome analysis**

Metabolites were extracted from 10 mg crushed, frozen liver tissue using 1 mL of cold methanol by vortexing for 1 min, followed by sonicating for 5 min. To precipitate protein, the methanol extracts were incubated on ice for 5 min. The extracts were then centrifuged at 16,000 ×*g* for 5 min at 4 °C, and the resultant supernatants were collected. The supernatant (200 µL) was mixed with 40 µL of methanol containing 0.66 nmol 10-camphorsulfonic acid, 2.0 nmol PC 15:0–18:1 (d_7_), 0.14 nmol PE 15:0–18:1 (d_7_), 0.4 nmol PS 15:0–18:1 (d_7_), 0.10 nmol PG 15:0–18:1 (d_7_), 0.4 nmol PI 15:0–18:1 (d_7_), 0.20 nmol PA 15:0–18:1 (d_7_), 0.90 nmol LPC 18:1 (d_7_), 0.040 nmol LPE 18:1 (d_7_), 5.0 nmol ChE 18:1 (d_7_), 4.0 nmol MG 18:1 (d_7_), 0.30 nmol DG 15:0–18:1 (d_7_), 0.70 nmol TG 15:0–18:1 (d_7_)–15:0, 0.40 nmol SM d18:1–18:1 (d_9_), 6.0 nmol cholesterol (d_7_), 0.4 nmol Cer d18:1 (d_7_)–15:0, 0.40 nmol HexCer d18:1 (d_7_)–18:1, and FA 16:0 (^13^C_16_) as internal standards for mass spectrometry-based metabolomics analysis. After mixing with 440 µL of chloroform and 352 µL of water, the aqueous and organic layers were separated by vortexing and subsequent centrifugation at 16,000 ×*g* and 4 °C for 5 min. The aqueous (upper) layer (600 µL) was transferred to a clean tube for IC/MS/MS and PFPP-LC/MS/MS analyses. After the aqueous layer extracts were evaporated under vacuum, the dried extracts were stored at −80 °C. Prior to IC/MS/MS and PFPP-LC/MS/MS analyses, the dried sample was reconstituted in 50 µL of water. The organic (lower) layer (200 µL) obtained using phase separation was placed in another tube. Finally, 200 µ L of methanol was added to the tube containing the organic lower later, which was stored at −80 °C until analysis. Lipidomic analysis was performed using C18-SFC/MS/MS and DEA-SFC/MS/MS.

**IC/MS analysis for anionic polar metabolites**

The IC (Dionex ICS-5000^+^ HPIC system, Thermo Fisher Scientific) was equipped with an anion electrolytic suppressor (Dionex AERS 500e, Thermo Fisher Scientific) to convert the potassium hydroxide gradient into pure water before the sample enters the Q Exactive mass spectrometer (Thermo Fisher Scientific). The IC conditions were as follows: columns, a Dionex IonPac AG11-HC-4 μm guard column (2 mm i.d. × 50 mm, 4 µm particle size, Thermo Fisher Scientific) and a Dionex IonPac AS11-HC-4 µm column (2 mm i.d. × 250 mm, 4µm particle size, Thermo Fisher Scientific); injection volume, 5 μL; column temperature, 30 °C; mobile phase, potassium hydroxide (1−100 mM); post-column make-up pump solvent, 1 mM ammonium acetate in methanol; flow rate of IC mobile phase, 0.3 mL/min; and flow rate of make-up pump, 0.1 mL/min. The potassium hydroxide gradient conditions were as follows: 10−100 mM, 0−24 min; 100 mM, 24−27 min; and 10 mM, 27−35 min. The full scanning HRMS analysis conditions were as follows: polarity, positive and negative ionization; sheath gas flow rate, 50 arb; auxiliary (Aux) gas flow rate, 10 arb; spray voltage for positive ion mode, 3.5 kV; spray voltage for negative ion mode; −2.0 kV; capillary temperature, 250 °C; S-lens level, 50; heater temperature, 400 °C; mass resolution, 70,000; automatic gain control (AGC) target, 1 × 10^6^; maximum injection time, 200 ms; and scan range, 70−1,050 (*m/z*). The conditions for data-dependent MS^2^ (dd-MS^2^) were as follows: mass resolution, 17,500; AGC target, 5 × 10^4^; trap fill time, 80 ms; isolation width, ± 1.2 Da; fixed first mass, *m/z* 50; stepped normalized collision energy, 10, 30, and 45 eV; intensity threshold of precursor ions for dd-MS^2^ analysis, 1 × 10^4^; apex trigger, 2−4 s; and dynamic exclusion, 2 s.

**PFPP-LC/MS/MS analysis for cationic polar metabolites**

The PFPP-LC (Nexera X2 UHPLC system, Shimadzu Co., Kyoto, Japan) conditions were as follows: column, a Discovery HS F5 column (2.1 mm i.d. × 150 mm, 3 µm particle size, Merck); injection volume, 2 μL; column temperature, 40 °C; mobile phase A, 0.1% formic acid in water; mobile phase B, acetonitrile; and flow rate, 0.25 mL/min. The gradient conditions were as follows: 0% B, 0−5 min; 0−40% B, 5−15 min; 40−100% B, 15−18 min; and 0% B, 18−25 min. The full scanning HRMS analysis conditions were as follows: sheath gas flow rate, 40 arb; capillary temperature, 350 °C; and heater temperature, 300 °C. The other full scanning HRMS and the dd-MS^2^ parameters were the same as those used in the IC/MS/MS analysis.

**DEA-SFC/MS/MS analysis for quantitative lipidomics**

The DEA-SFC (Nexera UC system, Shimadzu) conditions were as follows: column, an ACQUITY UPC^2^ Torus diethylamine (3.0 mm i.d. × 100 mm, 1.7 µm particle size, Waters, Milford, MA); injection volume, 2 μL; column temperature, 50 °C; mobile phase A, supercritical carbon dioxide; mobile phase B (modifier) and make-up pump solvent, methanol/water (95/5, v/v) with 0.1% (w/v) ammonium acetate; flow rate of mobile phase, 1.0 mL/min; flow rate of make-up pump, 0.1 mL/min; and back pressure regulator, 10 MPa. The gradient conditions were as follows: 1% B, 0−1 min; 1−75% B, 1−24 min; 75% B, 24−26 min; and 1% B, 26−30 min. The triple quadrupole mass spectrometer (TQMS, LCMS-8060, Shimadzu) analysis conditions were as follows: polarity, positive and negative ionization; electrospray voltage, 4 kV in the positive ion mode and ‒3.5 kV in the negative ion mode; nebulizer gas flow rate, 3.0 L/min; drying gas flow rate, 10.0 L/min; desolvation line temperature, 250 °C; heat block temperature, 400 °C; and detector voltage, 2.16 kV. The multiple reaction monitoring (MRM) parameters per time period were as follows: limit on number of MRM transitions, 150; dwell time, 2 ms; pause time, 2 ms; and polarity switching time, 5 ms. Other optimized MRM parameters for lipids (PCs, PEs, PSs, PGs, PIs, PAs, LPCs, LPEs, MGs, DGs, TGs, SMs, cholesterol, Cers, and HexCers) are shown in Table S7.

**C18-SFC/MS/MS analysis for determination of FAs and ChEs**

The SFC with an ACQUITY UPC^2^ HSS C18 SB column (3.0 mm i.d. × 100 mm, 1.8 µm particle size, Waters) gradient conditions were as follows: 0−50% B, 0−25 min; 50% B, 25−28 min; and 0% B, 28−30 min. Optimized MRM parameters for FAs and ChEs are shown in Table S7. The other C18-SFC and TQMS parameters were the same as those used in the DEA-SFC/MS/MS analysis.

**Sample preparation for proteome analysis**

The feces and livers of the male db/db mice (12-weeks-old) were collected and cut into small pieces using dissection scissors. For fecal samples, 450 µl methanol was added to 10 mg feces; then, 90 µl of the suspension was diluted with 450 µl methanol. Distilled water (250 µl) and 500 µl chloroform were added to the diluted suspension, followed by vortexing. After centrifugation at 4,600 ×*g* for 5 min, both the organic and aqueous phases were removed, and pellets in the interphase were dried under vacuum.

Proteins were extracted from the dried extracts of the feces and the disrupted livers using the phase-transfer surfactant method ^1^ with a slight modification. The samples were lysed using 100 mM Tris-HCl (pH 9.0) containing 12 mM sodium deoxycholate, 12 mM sodium lauroyl sarcosinate, and protease cocktails (Sigma), and protein concentration was determined using the bicinchoninic acid assay. Bovine serum albumin was used as the standard.

The extracted protein was subjected to reductive alkylation, followed by successive digestion using Lys-C endopeptidase and trypsin, as described previously ^2^. After acidification using trifluoroacetic acid (TFA), the surfactants were removed via ethyl acetate extraction, and the digested peptides in the aqueous fraction were desalted using a StageTip ^3^ cast with an SDB-XC Empore disk membrane (3M). The mouse liver digests were isotopically labeled using Tandem Mass Tag (TMT) 10-plex labeling kits ^4^ (Thermo Fisher Scientific) according to the manufacturer’s protocol and were desalted as described earlier. For parallel reaction monitoring (PRM) analysis, stable-isotope dimethyl labeling was performed, as previously described ^5^. The digests of the fecal samples and synthetic peptides were labeled using normal formaldehyde and ^2^H_2_,^13^C-formaldehyde, respectively, then mixed and desalted as described earlier.

**Nano LC/MS/MS for proteome analysis**

The digested peptides were analyzed using nanoLC/MS/MS, using an Orbitrap Fusion Lumos mass spectrometer (Thermo Fisher Scientific) in data-dependent acquisition (DDA) mode, or a Q Exactive mass spectrometer (Thermo Fisher Scientific) for PRM mode, coupled to an UltiMate 3000 RSLCnano system (CTC Analytics) and HTC-PAL autosampler (CTC). MonoCap C18 HighResolution 2000 columns (GL Sciences, Inc.), and a homemade column containing ReproSil C18 materials (3 μm, Dr. Maisch) were packed into a self‐pulled fused silica capillary (150 mm length × 100 μm I.D., 6 μm opening); these were used for DDA and PRM analysis, respectively. The mobile phases consisted of (A) 0.5% acetic acid and (B) 0.5% acetic acid and 80% acetonitrile. A two-step linear gradient of 5–40% B for 480 min, 40–100% B for 5 min, and 100% B for 10 min was used for DDA analysis. A two-step linear gradient of 5–40% B for 65 min, 40–100% B for 1 min, and 100% B for 4 min was used for PRM analysis. Spray voltage of 2,400 V was applied in both the Lumos and Q Exactive systems. DDA analysis using the Lumos system was performed in top-speed mode with a 3-s cycle time. The full MS scan was performed using a scan range of *m/z* 375−1,500, automatic gain control (AGC) target of 4 × 10^5^, and maximum injection time of 50 ms. The precursor ions were selected using an isolation window of 0.7 *m/z* in each MS scan for successive MS/MS scans, with collision-induced dissociation (CID) at normalized collision energy of 35%, target AGC of 1 × 10^4^, and maximum injection time of 100 ms; the fragment ions were scanned using an ion trap. For analyzing the TMT-labeled samples, synchronous precursor selection (SPS) ^6^ was performed using higher-energy collisional dissociation (HCD) at 10 SPS precursors, normalized collision energy of 65%, target AGC of 5 × 10^4^, and maximum injection time of 100 ms; the MS^3^ fragment ions were scanned using an Orbitrap mass spectrometer. The scan ranges of MS2 and MS3 were set to 400−1,200 and 100−500, respectively. The PRM analysis using the Q Exactive system was performed with a normalized HCD collision energy of 27, isolation window of 1.0 m/z, target AGC of 1 × 10^6^, and maximum injection time of 200 ms. The +2 and +3-charged ions of dimethylated unique *Pg* peptides were monitored as the precursor ions. The other settings were the same as described previously ^2,7^.

**Data processing for proteome analysis**

Database searching was performed as previously described ^2^, with a slight modification. Peak lists were generated from the raw DDA analysis data using ProteoWizard ^8^, on the basis of the recorded fragmentation spectra. For metaproteome analysis of mouse feces, peptides were identified via automated database searching, using Mascot v. 2.6.1 (Matrix Science, London), against a custom database. This custom database comprises the UniProtKB/ReferenceProteome (2017/11) of *Mus musculus, P. gingivalis,* the proteomes of four major food plants (*Oryza sativa*, *Glycine max*, *Triticum aestivum,* and *Zea mays*), and a database derived from a large-scale metagenome analysis of mouse gut ^9^. The *Mus musculus* UniProtKB/ReferenceProteome (2017/11) was used for analysis of mouse liver. Mass tolerance of 10 ppm and 0.8 Da was applied for precursor and fragment ions, respectively. Trypsin was specified as the reference enzyme, and up to two missed cleavages were allowed. Carbamidomethylation of cysteine was set as a fixed modification, and oxidation of methionine and TMT-labeling of amino groups at the peptide N-terminus and in lysine side chains were allowed as variable modifications. Microbiome profiling based on the metaproteome of the feces was performed using Unipept 4.0 ^10^.

**Supporting Information references**

1 Masuda, T., Tomita, M. & Ishihama, Y. Phase transfer surfactant-aided trypsin digestion for membrane proteome analysis. *J Proteome Res* **7**, 731-740, doi:10.1021/pr700658q (2008).

2 Sugiyama, N. *et al.* Comparative proteomics of Helicobacter pylori strains reveals geographical features rather than genomic variations. *Genes Cells* **24**, 139-150, doi:10.1111/gtc.12662 (2019).

3 Rappsilber, J., Mann, M. & Ishihama, Y. Protocol for micro-purification, enrichment, pre-fractionation and storage of peptides for proteomics using StageTips. *Nat Protoc* **2**, 1896-1906, doi:10.1038/nprot.2007.261 (2007).

4 Werner, T. *et al.* Ion coalescence of neutron encoded TMT 10-plex reporter ions. *Anal Chem* **86**, 3594-3601, doi:10.1021/ac500140s (2014).

5 Boersema, P. J., Raijmakers, R., Lemeer, S., Mohammed, S. & Heck, A. J. Multiplex peptide stable isotope dimethyl labeling for quantitative proteomics. *Nat Protoc* **4**, 484-494, doi:nprot.2009.21 [pii] 10.1038/nprot.2009.21 (2009).

6 McAlister, G. C. *et al.* MultiNotch MS3 enables accurate, sensitive, and multiplexed detection of differential expression across cancer cell line proteomes. *Anal Chem* **86**, 7150-7158, doi:10.1021/ac502040v (2014).

7 Tajima, K. *et al.* Mitochondrial lipoylation integrates age-associated decline in brown fat thermogenesis. *Nat Metab* **1**, 886-898, doi:10.1038/s42255-019-0106-z (2019).

8 Kessner, D., Chambers, M., Burke, R., Agus, D. & Mallick, P. ProteoWizard: open source software for rapid proteomics tools development. *Bioinformatics* **24**, 2534-2536, doi:10.1093/bioinformatics/btn323 (2008).

9 Xiao, L. *et al.* A catalog of the mouse gut metagenome. *Nat Biotechnol* **33**, 1103-1108, doi:10.1038/nbt.3353 (2015).

10 Gurdeep Singh, R. *et al.* Unipept 4.0: Functional Analysis of Metaproteome Data. *J Proteome Res* **18**, 606-615, doi:10.1021/acs.jproteome.8b00716 (2019).

**Supplementary Table** **S1.** Peptides identified by metaproteome analysis of mouse feces.

^a^CMC 1 day: CMC control-treated, 1 d after 1^st^ administration

^b^*Pg* 1 day: *Pg*-treated, 1 d after 1^st^ administration

^c^CMC 30 days: CMC control-treated, 3 d after 10^th^ administration

^d^*Pg* 30 days: *Pg*-treated, 3 d after 10^th^ administration. *Pg*: *Porphyromonas gingivalis*.

Supplementary Table S2 is shown in Supplementary Dataset.

**Supplementary Table** **S2.** PCR primers used in this work.

| **Primer list** | | |  | |  |  |
| --- | --- | --- | --- | --- | --- | --- |
| Gene name | Forward | | | Reverse | | |
| Phosphoenolpyruvate carboxykinase 1 (Pck1) | | CCATCCCAACTCGAGATTCTG | | CTGAGGGCTTCATAGACAAGG | | |
| Glucose-6-phosphatase, catalytic (G6pc) | | TCTTGTGGTTGGGATTCTGG | | CGGATGTGGCTGAAAGTTTC | | |
| Forkhead box protein O1 (Foxo1) | | CTACGAGTGGATGGTGAAGAG | | TGTGAAGGGACAGATTGTGG | | |
| Carnitine palmitoyltransferase I (Cpt1c) | | GCCCAGTATGAGAGGATGTTC | | TCTTGAAGGTGACGAAGGTG | | |
| Fatty acid synthase (Fasn) | | CCCCTCTGTTAATTGGCTCC | | TTGTGGAAGTGCAGGTTAGG | | |
| acetyl-Coenzyme A carboxylase alpha (Acaca) | | AAGGCTATGTGAAGGATGTGG | | CTGTCTGAAGAGGTTAGGGAAG | | |
| Sterol regulatory element-binding transcription factor 1 (Srebf1) | | CCATCGACTACATCCGCTTC | | GCCCTCCATAGACACATCTG | | |
| Sterol regulatory element binding transcription factor 2 (Srebf2) | | CCCTATTCCATTGACTCTGAGC | | CACATAAGAGGATTCGAGAGCG | | |
|  | |  | |  | | |
| Interleukin 6 (Il-6) | | CCACTTCACAAGTCGGAGGCTTA | | GCAAGTGCATCATCGTTGTTCATAC | | |
| Tumor necrosis factor (Tnf-α) | | AAGCCTGTAGCCCACGTCGTA | | GGCACCACTAGTTGGTTGTCTTTG | | |
| Chemokine (C-C motif) ligand 2 (Ccl2) | | GCATCCACGTGTTGGCTCA | | CTCCAGCCTACTCATTGGGATCA | | |
| Chemokine (C-X-C motif) ligand 10 (Cxcl10) | | TGAATCCGGAATCTAAGACCATCAA | | AGGACTAGCCATCCACTGGGTAAAG | |  |
|  | |  | |  | |  |
| 18S ribosomal RNA (18Sr) | | TTCTGGCCAACGGTCTAGACAAC | | CCAGTGGTCTTGGTGTGCTCA | |  |

**Supplementary Tables S3-7 are shown in Supplementary Dataset.**

**Supplementary Table S3.** Protein fold changes and peptides identified by proteome analysis of *Porphyromonas gingivalis* (*Pg*)- or CMC-treated *db/db* mice livers.

Three biological replicates and three technical replicates were included for each value.

**Supplementary Table S4.** Hydrophilic metabolite profiles of the small intestines of *Porphyromonas gingivalis* (*Pg*)-treated *db/db* mice.

**Supplementary Table S5.** Hydrophilic metabolite profiles of livers.

**Supplementary Table S6.** Hydrophobic metabolite profiles of livers.

**Supplementary Table S7.** Enzyme expression associated with each metabolic reaction.

**Supplementary Fig. S1.** PRM analysis of the six distinct peptides derived from *Porphyromonas gingivalis*. The sample preparation was performed in triplicate from the pooled feces and the peptides were quantified based on median of peak area ratios of each transition. Corresponding synthetic peptides were used as standard.

**Supplementary Fig. S2.** Intrahepatic lipid profiling of *Porphyromonas gingivalis* (*Pg*)*-* and control-treated *db/db* mice. Comparison of absolute quantities of lipid classes between *Pg*- and control-treated mice. Black bars: CMC (control)-treated. Red bars: *Pg*-treated. Lipid contents did not differ between the *Pg*- and control-treated mice.

**Supplementary Fig. S3.** Effects of *Porphyromonas gingivalis* (*Pg*) LPS (1μg/ml) (Invivogen) or human recombinant IL-1β (10 ng/ml) (invitrogen) on the mRNA expression of IL-6, CCL2, CXCL10 and FOXO1 in HepG2 cells. mRNA expression was determined using quantitative RT-PCR and was normalized against the expression of 18S rRNA mRNA. Target gene expression in *Pg* LPS or IL-1β -treated groups were normalized against the target gene expression in control group, which is considered as 1. in every chart. n = 3, * *P* < 0.05 vs control.
