# Supplementary material for: Porphyromonas gingivalis induces entero-hepatic metabolic derangements with alteration of gut microbiota in a type 2 diabetes mouse model
Source: Sci Rep. 2021 Sep 15;11:18398. doi: 10.1038/s41598-021-97868-2 (PMC8443650; doi:10.1038/s41598-021-97868-2)
Supplement: Supplementary file 5 — Supplementary Information 5. [file 41598_2021_97868_MOESM5_ESM.pptx]

## Slide 1
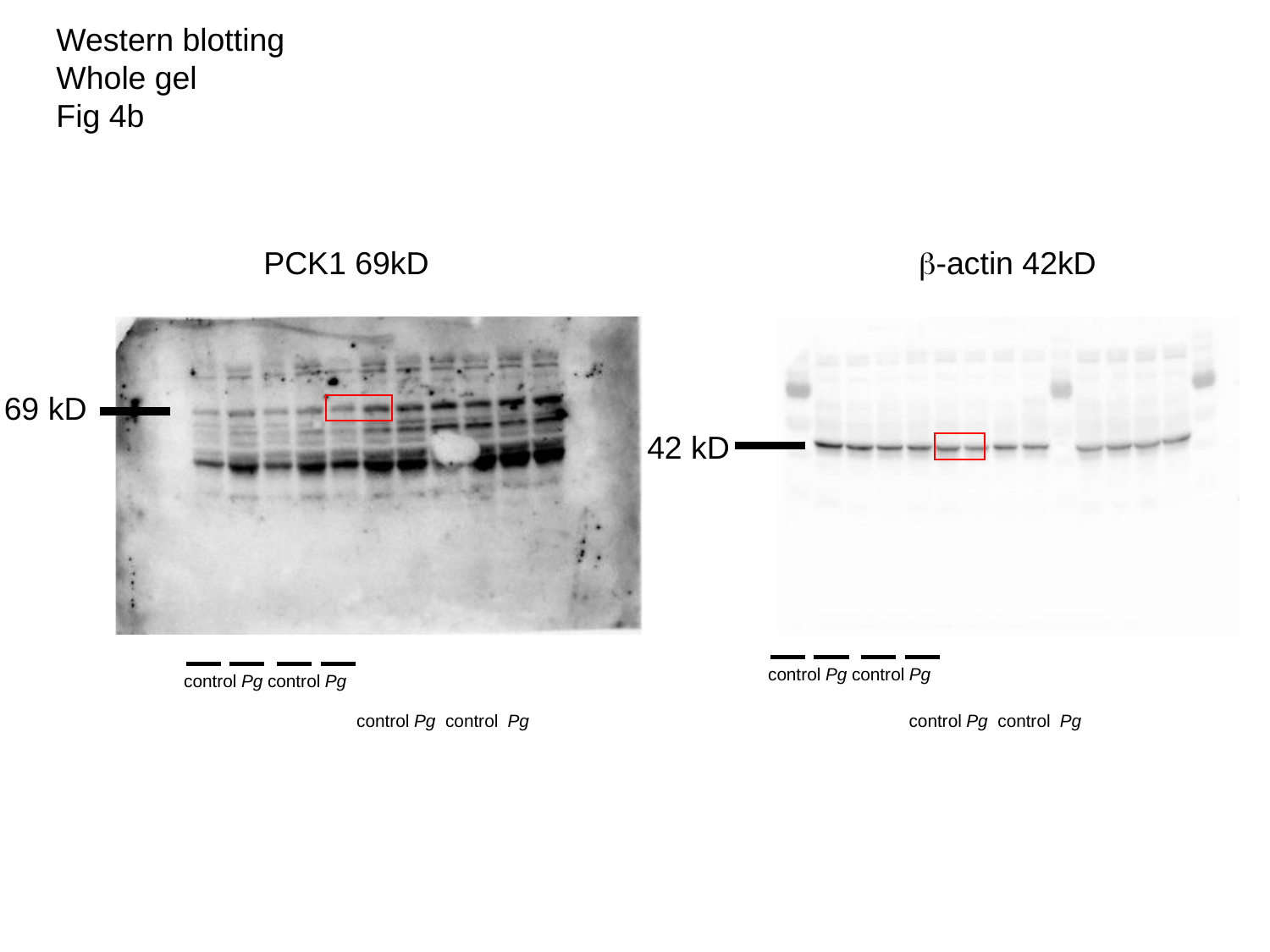

Western blotting
Whole gel
Fig 4b
PCK1 69kD
b-actin 42kD
69 kD
42 kD
control Pg control Pg
control Pg control Pg
control Pg control Pg
control Pg control Pg

## Slide 2
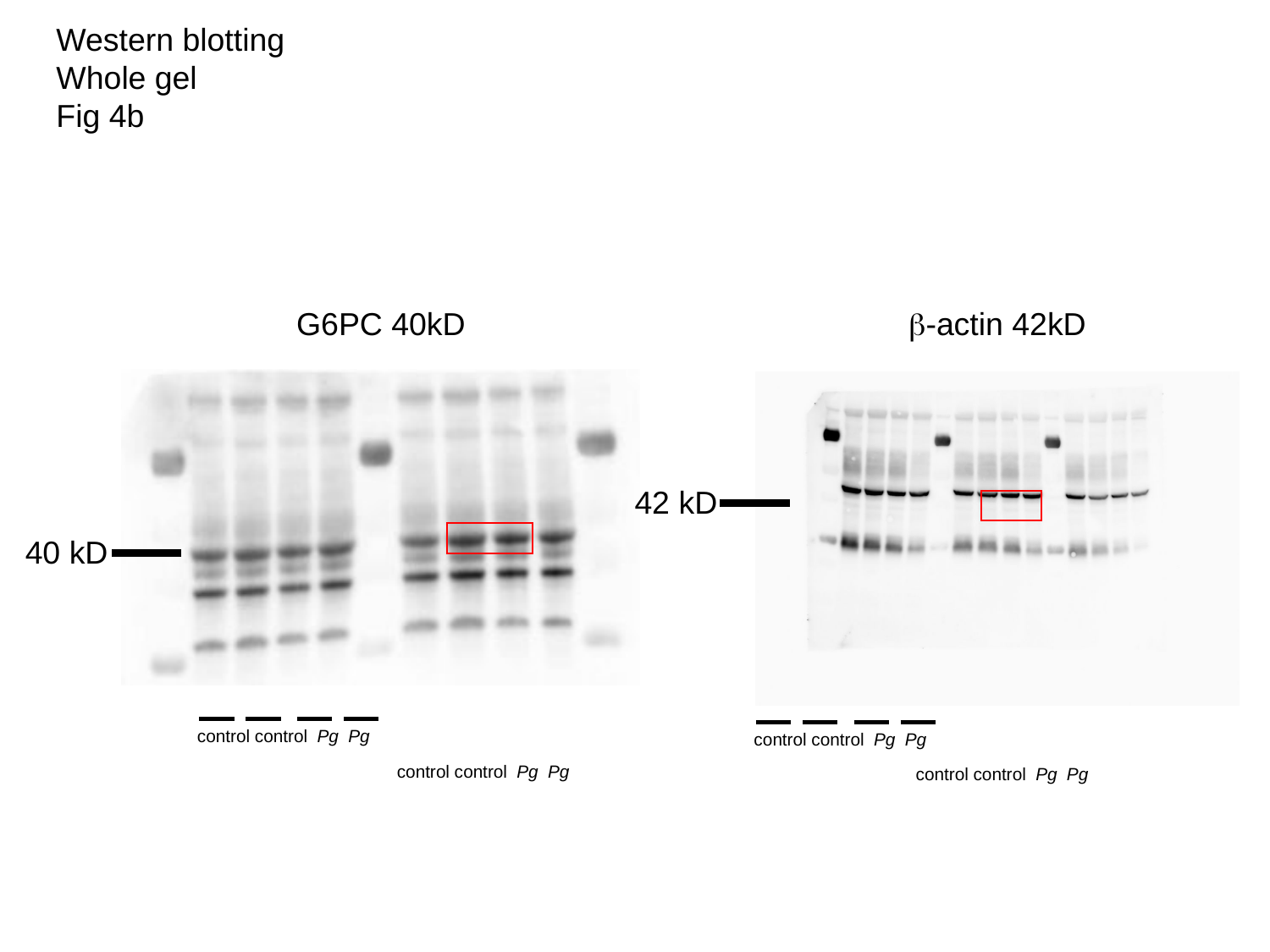

Western blotting
Whole gel
Fig 4b
G6PC 40kD
b-actin 42kD
42 kD
40 kD
control control Pg Pg
control control Pg Pg
control control Pg Pg
control control Pg Pg

## Slide 3
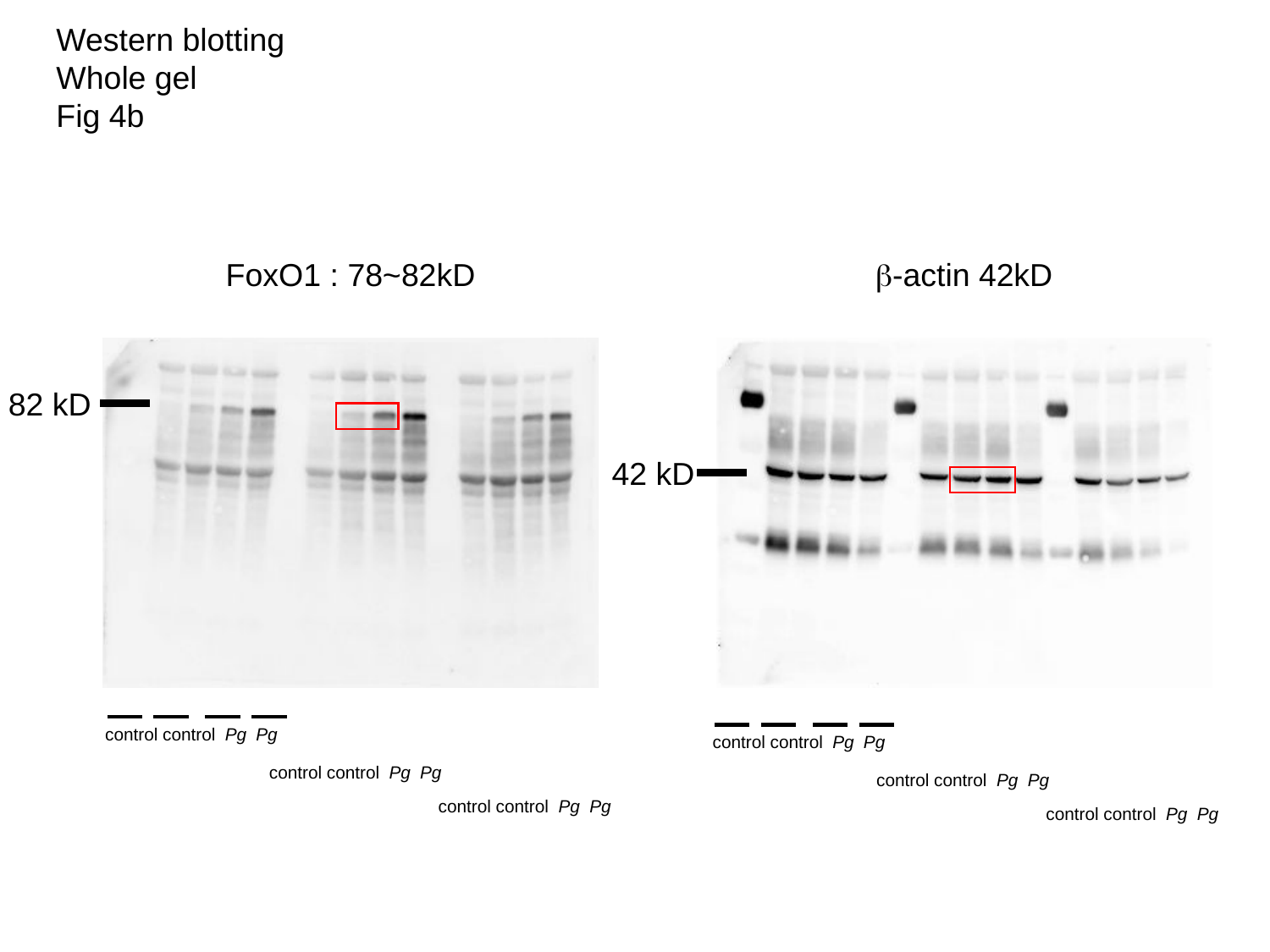

Western blotting
Whole gel
Fig 4b
FoxO1 : 78~82kD
b-actin 42kD
82 kD
42 kD
control control Pg Pg
control control Pg Pg
control control Pg Pg
control control Pg Pg
control control Pg Pg
control control Pg Pg
